# Supplementary material for: Single Cell Transcriptomic and Chromatin Profiles Suggest Layer Vb Is the Only Layer With Shared Excitatory Cell Types in the Medial and Lateral Entorhinal Cortex
Source: Front Neural Circuits. 2022 Jan 26;15:806154. doi: 10.3389/fncir.2021.806154 (PMC8826650; doi:10.3389/fncir.2021.806154)
Supplement: Supplementary file 14 [file Data_Sheet_1.ZIP › Deep_vs_Sup_Motifs/homerResults/motif8.info.html]

Motif 8

## Information for 14-YAGGAATC (Motif 8)

A
G
C
T
C
G
T
A
A
C
T
G
C
T
A
G
C
G
T
A
C
G
T
A
A
C
G
T
A
G
T
C
  
Reverse Opposite:
